# Superconductivity and Shubnikov - de Haas effect in polycrystalline Cd_3_As_2_ thin films

**DOI:** 10.1038/s41598-020-61376-6

**Published:** 2020-03-12

**Authors:** Leonid N. Oveshnikov, Alexander B. Davydov, Alexey V. Suslov, Alexey I. Ril’, Sergey F. Marenkin, Alexander L. Vasiliev, Boris A. Aronzon

**Affiliations:** 1 National Research Center “Kurchatov Institute”, Moscow, 123182 Russia; 2 P. N. Lebedev Physical Institute, Russian Academy of Sciences, Moscow, 119991 Russia; 3 National High Magnetic Field Laboratory, Tallahassee, Florida 32310 USA; 4 Kurnakov Institute of General and Inorganic Chemistry, Russian Academy of Sciences, Moscow, 119991 Russia; 5 National University of Science and Technology “MISiS”, Moscow, 119049 Russia; 6 Moscow Institute of Physics and Technology, Dolgoprudny, Moscow region 141700 Russia

**Keywords:** Topological matter, Surfaces, interfaces and thin films, Superconducting properties and materials

## Abstract

In this study we observed the reproducible superconducting state in Cd_3_As_2_ thin films without any external stimuli. Comparison with our previous results reveals similar qualitative behavior for films synthesized by different methods, while the difference in the values of the critical parameters clearly shows the possibility to control this state. The X-ray diffraction measurements demonstrate the presence of the tetragonal Cd_3_As_2_ crystal phase in studied films. Measurements of high-field magnetoresistance reveal pronounced Shubnikov - de Haas oscillations. The analysis of these oscillations suggests that, due to high carrier concentration in studied Cd_3_As_2_ films, the initial Dirac semimetal phase may be partially suppressed, which, however, does not contradict with possible topological nature of the observed superconductivity.

## Introduction

Recently Dirac materials became one of the most intensively studied areas of condensed matter physics. During the past two decades many efforts were directed towards the experimental and theoretical studies in this emerging field, with focus on low-dimensional Dirac systems (such as graphene and topological insulators). Nowadays more attention is paid to the systems with three-dimensional Dirac spectrum - Dirac and Weyl semimetals (DSM and WSM)^1,2^. The WSM system posses even number of isolated points in the bulk Brillouin zone (Weyl nodes), where conductance and valance bands touch, forming in its vicinity states with linear dispersion and fixed mutual orientation of momentum and spin directions (spin chirality), which results in non-zero Berry curvature of bulk spectrum and unique surface states. By restoring the inversion or time-reversal symmetry one can tune such system into DSM phase resulting in the two-fold chiral degeneracy of electron states (near Dirac nodes)^1,2^. Among several candidates to host DSM phase, Cd_3_As_2_ appears to be one of the most promising ones, due to its air stability, relatively simple band structure, and outstanding transport properties^3–5^. It was experimentally shown that magnetoresistance oscillations in Cd_3_As_2_ experience a phase shift related to the Berry phase^6^, as it is suggested for the DSM. Moreover, Cd_3_As_2_ thin films served as a platform for the realization of quantum Hall state^7,8^ and allowed studying properties of unconventional surface states^9,10^. While the fundamental characteristics of DSM systems are of great interest, for the application purposes one needs to incorporate certain additional properties into DSM material, such as magnetism or superconductivity (SC).

Theoretical studies revealed that in Cd_3_As_2_ intrinsic superconductivity can emerge, and by varying the parameters of the inter- and intraorbital $e-e$ attractions in this system, the pairing potential can change its parity from even to odd, realizing topological SC (TSC) state in the latter case^11^. Such TSC paves a way to realizing Majoranna modes, e.g., required in quantum computing^12^. However, the actual observation of TSC in real materials is still under debate. Though the SC state in Cd_3_As_2_ was observed experimentally and claimed to have topological nature, most of related studies dealt with somewhat artificial SC driven by hydrostatic^13^ or local pressure^14–16^ or induced via the proximity effect with a conventional superconductor^17–19^. As a result, for interpretation of such data one needs to take into account various effects related to the symmetry lowering due to crystal deformation or to the details of heterogeneous systems. It is also worth mentioning, that the majority of these studies were limited to small magnetic fields, where the SC state was observed, while they lacked data on the conservation of various features typical for native DSM phase, thus, questioning the actual reasons for the TSC to occur.

In our previous study we discovered SC in magnetron-sputtered Cd_3_As_2_ films^20^ which revealed several features indicating possible topological nature of the observed state. To exclude possible effects related to specific details of the chosen synthesis technique in the study reported there, we investigated similar films produced by a different method and observed a reproducible SC state, showing similar qualitative behavior. Thus, we argue that SC is the universal feature of polycrystalline Cd_3_As_2_ films. Moreover, both (i) presence of Cd_3_As_2_ tetragonal crystalline phase and (ii) Shubnikov - de Haas oscillations observed in high magnetic fields suggest preservation of the DSM phase in studied films. We propose such polycrystalline thin films as a promising playground to study superconducting DSM systems.

## Results and discussion

**Figure 1:**
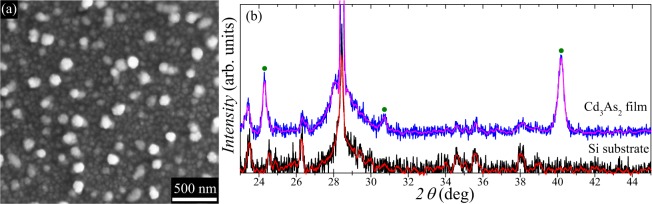
(**a**) Typical secondary electron SEM image of the studied films surface. (**b**) XRD pattern for studied Cd_3_As_2_ sample mainly reproduces the pattern for the (111) Si substrate except for three peaks marked by green circles, which correspond to the Cd_3_As_2_ crystal phase (see text). Red and magenta lines are smoothed data presented for clarity. The experiment was conducted using a sample with a Hall bar mesa which explains the substantial signal related to the substrate.

Scanning electron microscope (SEM) study of both specimens under investigations revealed similar surface morphology features (Fig. 1a). Isolated equiaxed grains with lateral dimensions in the range between 100 and 150 nm and having bright contrast are visible on the surface. The energy-dispersive X-ray (EDX) spectroscopy results demonstrated that the Cd and As distributed homogeneously within the film, while the actual elemental composition is close to the stoichiometric Cd_3_As_2_ within 2% accuracy (corresponding results include both averaging over large surface areas and analysis in points with different contrast). Described SEM results are very close to those reported earlier for magnetron-sputtered f ilms^20^. The crystal structure of studied films were investigated using X-ray diffraction (XRD) measurements (see Fig. 1b). In this work, for the XRD and transport measurements we used similar samples with a film shaped as a Hall bar. Thus, the studied film covered only a part of the investigated sample surface, which resulted in the substantial XRD signal related to the substrate. Therefore, the obtained XRD pattern for the sample under study mainly reproduces the pattern for (111) Si substrate with a native oxide layer (Fig. 1b). However, one can distinguish three pronounced peaks (marked by green circles in Fig. 1b) at $2\theta \approx 24.{3}^{\circ }$, $30.{7}^{\circ }$, and $40.{1}^{\circ }$ related to the film itself. It is worth mentioning that Cd_3_As_2_ crystals have three polymorphic modifications with the tetragonal lattice^21^: $\alpha$ (s.g. $I{4}_{1}$/$acd$, $a=12.6461$ Å, $c=25.4378$ Å), $\alpha {\rm{{\prime} }}$ (s.g. $P{4}_{2}$/$nbc$, $a=12.6848$ Å, $c=25.4887$ Å) and $\alpha {\prime\prime}$ (s.g. $P{4}_{2}$/$nmc$, $a=9.0364$ Å, $c=12.6606$ Å). In our previous study of Cd${}_{3}$As${}_{2}$ films synthesized by magnetron sputtering^20^, we argued that the polycrystalline nature of samples was partially related to the presence of both $\alpha$ and $\alpha {\rm{{\prime} }}$ phases. Corresponding XRD patterns also indicated partial orientation of Cd_3_As_2_ crystallites with the average size of about 35 nm. In this study we observed three peaks (Fig. 1b) corresponding to the most intensive peaks in powder XRD patterns for all tetragonal phases of Cd_3_As_2_. On the one hand, the fact that the ratios between intensities of the observed peaks are close to relative intensities of the Cd_3_As_2_ powder reference implies the absence of any pronounced Cd_3_As_2_ crystallites orientation in studied films. On the other hand, as only three XRD peaks were detected it was not possible to unambiguously determine the exact tetragonal modification of Cd_3_As_2_ lattice in our samples. The latter seems to be not so crucial as, to the best of our knowledge, there is no clear evidence that the DSM state is restricted only to any specific tetragonal phase of Cd_3_As_2_. Using the standard Debye-Scherrer equation with the shape factor of 0.9, we estimated the mean crystallite size to be $D\,\approx \,$20–25 nm.

**Figure 2:**
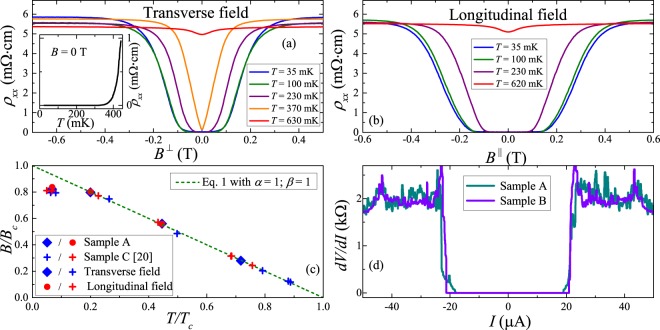
Low-field magnetoresistance of sample A at various temperatures for (**a**) transverse and (**b**) longitudinal magnetic field orientations. Panel (a) inset: temperature dependence of sample’s A resistivity in the low-temperature region of the SC transition. (**c**) ${B}_{c}-{T}_{c}$ diagram in reduced coordinates (${B}_{c}^{\perp }=0.2$ T, ${T}_{c}=520$ mK, ${B}_{c}^{\parallel }/{B}_{c}^{\perp }\approx 1.7$). Dashed line - approximation by Eq. (1) with $\alpha =1;\beta =1$. For comparison we also provide corresponding data for the magnetron-sputtered film (Sample C from our previous study^20^). (**d**) Differential resistance for samples A and B at $T\approx 30$ mK.

Transport measurements reveal that resistances of our samples are drastically dropping down to zero at temperatures below 1 K thus revealing SC transitions. A temperature dependence ${\rho }_{xx}(T)$ in a low temperature domain of the SC transition is plotted in the inset in Fig. 2a and clearly shows the zero resistance value in the SC state. Note that the superconducting state in the films extends above 0.3 K, i.e., above the temperature where a superconductive transition was observed in our magnetron-sputtered films^20^. We also observed corresponding features of low-field magnetoresistance (Fig. 2a) related to the field-induced transition into the normal state. The observed low-field dip becomes narrower and eventually disappears along with the temperature increase, which is also a typical behavior of SC systems. In the longitudinal field (Fig. 2b) this dip becomes substantially wider indicating higher critical field, ${B}_{c}$, values, which is characteristic for SC thin films. Following the arguments described earlier^20^, we estimated ${B}_{c}$ values from the 50% drop of the sample resistivity. We consider obtained ${B}_{c}-{T}_{c}$ diagrams (Fig. 2c) using the conventional formula:

1 $$B(T)={B}_{c}(0)\cdot {\left(1-{\left(\frac{T}{{T}_{c}}\right)}^{\alpha }\right)}^{\beta }.$$

Where, $\alpha =2;\beta =1$ corresponds to the Bardeen-Cooper-Schrieffer theory and $\alpha =1;\beta =1$ - to the Ginzburg-Landau (G-L) theory. We should note that samples A and B show the same behavior with close SC parameters values (e.g., the difference in ${B}_{c}$ values is below 10%). Similar to our previous study^20^, investigated films reveal a linear ${B}_{c}-{T}_{c}$ region at an intermediate temperature range, suggesting the applicability of G-L theory. However, ${B}_{c}$ saturates at the lowest temperatures. Moreover, this behavior does not depend on $B$ orientation, as both data sets coincide when plotted in reduced coordinates (Fig. 2c). Thus, the qualitative features of the SC state in studied films are very similar to those observed earlier^20^, including exactly the same character of the ${B}_{c}-{T}_{c}$ diagram (for comparison we also provide data for the magnetron-sputtered film in the Fig. 2c). However, we should mention that the actual values of SC parameters are substantially different in these two cases. First of all, we observe much higher critical field values: ${B}_{c}^{\perp }=0.165$ T in the saturation region and ${B}_{c}^{\perp }=0.200$ T obtained from Eq. (1) linear ($\alpha =1;\beta =1$) extrapolation to zero temperature, while for magnetron-sputtered films we observed ${B}_{c}^{\perp }\approx$ 0.027 T and 0.035 T, correspondingly. Higher critical fields are also accompanied by higher ${T}_{c}$ values $-0.52$ K in this study against 0.2–0.3 K reported earlier^20^. We used obtained ${B}_{c}^{\perp }$ values to estimate the coherence length $\xi$ in the framework of the G-L theory (${B}_{c}(0)={\phi }_{0}$/$(2\pi {\xi }^{2})$, ${\phi }_{0}$ - magnetic flux quantum). We obtained $\xi \approx 40$ nm, which is smaller than the film thickness ($\sim 100$ nm), unlike in our previous study. This finding also agrees with almost two times smaller critical field anisotropy (${B}_{c}^{\parallel }$/${B}_{c}^{\perp }\approx 1.7$), suggesting that studied films are closer to the 3D limit than to the 2D case. As an additional indication of SC nature of the observed behavior we investigated the differential resistance, $dV$/$dI$, of studied films, which also showed a typical shape with a zero-resistivity plateau (Fig. 2d). As one can see, the edges of zero-resistivity regions correspond to the critical current values of ${I}_{c}\approx 20$ *µ*A, which is slightly lower than reported earlier at same temperatures (about 25 $\mu$A). Similar qualitative features of the SC state in the films investigated here and those studied earlier^20^ suggest that the origin of SC can be the same in these two cases, while the significant difference in the values of SC parameters shows that this state can be effectively controlled.

**Figure 3:**
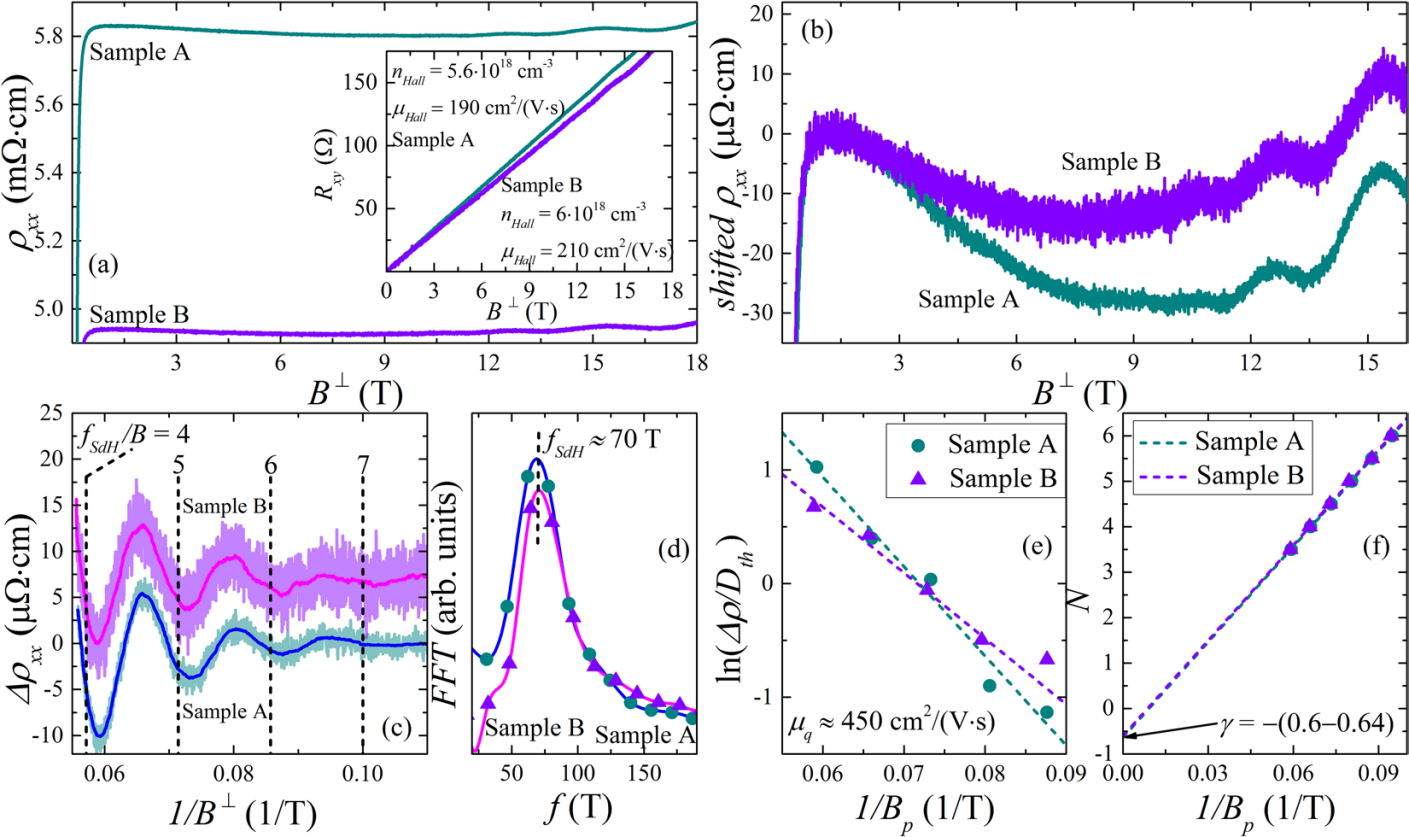
(**a**) High-field MR of studied samples at $T=0.29$ K. Panel (a) inset: Hall resistivity curves and corresponding Hall parameters for studied samples at $T=0.29$ K. (**b**) Enlarged MR curves, converged at lower fields, shown for clarity. One can see non-monotonic character of MR and distinguishable SdH oscillations. (**c**) SdH oscillations for studied samples after subtracting background MR. Due to relatively high noise, smoothed curves (blue and magenta) are also provided. Vertical lines correspond to the integer values of ${f}_{SdH}/B$ ratio for ${f}_{SdH}\approx 70$ T, obtained from corresponding Fourier spectra shown in panel (d). (**e**) Dingle plot for oscillations shown in panel (c). (**f**) Corresponding Landau fan diagram with linear approximation and marked phase factor $\gamma \approx -(0.6-0.64)$ (see text).

The aforementioned presence of a crystalline Cd_3_As_2_ phase suggests that along with a SC state the DSM phase should also be present in studied films. To investigate this possibility we measured the high-field transverse magnetoresistance (MR) (Fig. 3a). While the measured normal-state resistivity is somewhat different for samples A and B, they have a similar non-monotonic character of MR at higher field (Fig. 3b). Obtained Hall resistivity curves (see inset in Fig. 3a) suggest that lower normal-state resistivity for Sample B corresponds to slightly higher values of both carrier concentration and mobility. It should be mentioned that obtained ${n}_{Hall}$ values are close to the Hall concentrations for magnetron-sputtered films reported earlier^20^. Both samples also reveal Shubnikov - de Haas (SdH) oscillations (Fig. 3c), which are commonly used to characterize Dirac carriers in DSM^22–24^ and WSM^25^ systems. Corresponding Fourier spectra (Fig. 3d) show single frequency ${f}_{SdH}\approx 70$ T for both samples. Due to relatively high thickness of studied films it is natural to assume that the corresponding electron spectrum is three-dimensional. The Fermi surface of electrons in Cd_3_As_2_ single crystals has a weakly anisotropic ellipsoidal shape^22–24^, except for the vicinity of Lifshitz transition, where two Dirac cones merge^24^. Thus, for simplicity one can consider this Fermi surface as a spherical one. It is even more suitable in our case, given the polycrystalline character of studied films and the absence of pronounced crystallite orientation. Thus, obtained cross-sectional area of the Fermi surface ${A}_{F}={f}_{SdH}(2\pi e/\hslash )\approx 0.67$ nm${}^{-2}$ ($\hslash$ - is the Plank constant; $e$ - is the electron charge) corresponds to the Fermi wave vector ${k}_{F}\approx 0.46$ nm${}^{-1}$. However, to calculate electron concentration, ${n}_{SdH}$, the degeneracy factor, $\varepsilon$, is required, which is argued to be $\varepsilon =4$ (due to double valley and chirality degeneracy^26^) and $\varepsilon =2$ below and above the Lifshitz point, correspondingly. Unfortunately, existing literature contains rather scarce values of critical electron density corresponding to this transition - from $2\cdot 1{0}^{18}$ cm^-3^ ^24^ to $2\cdot 1{0}^{20}$ cm^-3^ ^27^. In our previous study of Cd${}_{2.979}$Zn${}_{0.021}$As${}_{2}$ single crystals^28^ we observed anisotropic SdH oscillations, which had single frequency of about 28 T in the transverse field and two close frequencies in the longitudinal field. Such behavior was ascribed to the nesting of two Fermi ellipsoids, i.e., to the Fermi level being close to the Lifshitz point. While Zn-doping varies the band structure of Cd_3_As_2_ crystal, we believe that due to low Zn content in the studied crystal^28^, we can use obtained ${f}_{SdH}\approx 28$ T value as an estimate of the Lifshitz transition in pure Cd_3_As_2_. The latter implies that ${f}_{SdH}\approx 70$ T for the films studied here corresponds to the Fermi level being above the Lifshitz point, i.e., $\varepsilon =2$. Thus, we obtain ${n}_{SdH}\approx 3.3\cdot 1{0}^{18}$ cm${}^{-3}$, which is a typical value for bulk Cd${}_{3}$As${}_{2}$^22,28,29^. It also yields integral electron mobility values in studied films of about $\mu \approx 350$ cm${}^{2}$/(V$\cdot$s). It is important to note that conducting electrons in Cd_3_As_2_ with Fermi level above the Lifshitz point should retain some DSM features, as the Berry curvature remains non-zero even above Lifshitz transition^30^. To further process our data we use the conventional Lifshitz-Kosevich theory^31^, in which an oscillating part of a MR is given by

2 $$\Delta \rho \propto {D}_{th}\cdot \exp \left(-\frac{\pi }{{\mu }_{q}B}\right)\cdot \cos 2\pi \left(\frac{{f}_{SdH}}{B}+\gamma \right),$$

where temperature damping factor is

3 $${D}_{th}=\frac{\frac{2{\pi }^{2}{k}_{B}T{m}^{\ast }}{\hslash eB}}{\sinh \left(\frac{2{\pi }^{2}{k}_{B}T{m}^{\ast }}{\hslash eB}\right)}.$$

Here ${\mu }_{q}$ - is a quantum mobility; $\gamma$ - is a phase factor (see below); ${k}_{B}$ - is the Boltzman constant; ${m}^{\ast }$ - is an electron effective mass. In principle, Eq. (2) can be used to fit experimental oscillations. However to reduce a number of variable parameters it is usually more convenient to determine them separately. For example, to estimate ${\mu }_{q}$ values one may use Dingle plot, stemming from the apparent relation

4 $${\rm{ln}}\left(\frac{\Delta \rho }{{D}_{th}}\right)\propto -\frac{\pi }{{\mu }_{q}{B}_{p}},$$

where ${B}_{p}$ values are the positions of oscillations extrema. Corresponding Dingle plots for studied samples (Fig. 3e) with ${m}^{\ast }=0.045{m}_{e}$^29^ (${m}_{e}$ - free electron mass) yield ${\mu }_{q}\approx 450$ cm${}^{2}$/(V$\cdot$s) (which can be recalculated into quantum lifetime ${\tau }_{q}={\mu }_{q}{m}^{\ast }$/$e=1.15\cdot 1{0}^{-14}$ s and Dingle temperature ${T}_{D}=\hslash$/$(2\pi {k}_{B}{\tau }_{q})=106$ K). It is important to mention that obtained ${\mu }_{q}$ values exceed integral mobilities $\mu$ (and Hall mobilities ${\mu }_{Hall}$), clearly indicating the presence of considerable inhomogeneities in studied films, that is manifested as a spatial mobility variations. Given the difference in ${n}_{Hall}$ and ${n}_{SdH}$ values these variations can also be accompanied by the Fermi energy fluctuations. The latter is applicable only if we consider the system being strictly above Lifshitz transition, otherwise we use $\varepsilon =4$ and obtain ${n}_{SdH}\approx {n}_{Hall}$, which suggests homogeneous carrier density within the film. In general, one can estimate local mobility in the high-mobility regions from the onset of SdH oscillations, that in our case gives ${\mu }_{loc}\ge 1000$ cm${}^{2}$/(V$\cdot$s). It is worth noting that ${\mu }_{loc}\gg {\mu }_{q}$ implies the dominant role of small-angle scattering in these high-mobility regions. Due to the absence of strictly formulated $E(k)$ relation for Cd_3_As_2_, we had to use simplified expressions in order to estimate characteristic Fermi energies for studied films. Thus, if we assume strictly linear spectrum with Fermi velocity ${\upsilon }_{F}=1.5\cdot 1{0}^{6}$ m/s^4^, then we obtain ${E}_{F}=\hslash {k}_{F}{\upsilon }_{F}\approx 450$ meV, while quantum coherent length of carriers will be ${l}_{q}={\upsilon }_{F}{\tau }_{q}=17.3$ nm. One the other hand, using simple parabolic dispersion we arrive at ${E}_{F}={\hslash }^{2}{k}_{F}^{2}$/$(2{m}^{\ast })\approx 180$ meV, ${\upsilon }_{F}=\hslash {k}_{F}$/${m}^{\ast }=1.2\cdot 1{0}^{6}$ m/s and ${l}_{q}=13.7$ nm.

Now we will address the last term in Eq. (2). The phase factor,$\gamma$, should be considered as a sum of several terms

5 $$\gamma =\frac{1}{2}+{\phi }_{B}+\delta .$$

The first term stems from the Onsager quantization rule; ${\phi }_{B}$ represents the Berry phase, which should be ${\phi }_{B}=-1$/2 for Dirac carriers and ${\phi }_{B}=0$ for trivial ones; $\delta =\pm$1/8 is related to 3D character of the Fermi surface in our case. The analysis of the SdH oscillations phase is widely used to investigate the properties of Dirac materials^6,22–24,32–34^. If one ascribes integer values of Landau level numbers, $N$, to the maxima of SdH oscillations, then the intercept of the linear approximation with the $N$ axis of Landau fan diagram ($N$ versus 1/${B}_{p}$ plot) should give the $\gamma$ value (which determines relative position of oscillation extrema and integer values of ${f}_{SdH}$/$B$ ratio, see Fig. 3c). Following this procedure we obtain $\gamma \approx -(0.6-0.64)$ for studied samples (Fig. 3f), which is close to the theoretical value of $\gamma =-5$/8 for the Cd_3_As_2_ with Fermi level above the Lifshitz point^33^. The latter implies ${\phi }_{B}=0$ due to the cancellation of phase shifts given by each Dirac node, when two ellipsoids merge. In addition, it was argued earlier that the ${\phi }_{B}\ne 0$ for Cd_3_As_2_ persists only for specific field orientations relative to the crystal axes, due to the transition into Weyl semimetal phase in high magnetic fields^23^. Due to the absence of pronounced Cd_3_As_2_ crystallites orientation in studied films, averaging over all relative orientations of the magnetic field may yield an average ${\phi }_{B}=0$ observed in our experiment. However, in this case, in the low field region, where superconductivity is observed, the DSM phase is preserved. Thus, the obtained results suggest that the studied SC Cd_3_As_2_ films host the DSM phase, which may be partially suppressed due to the Lifshitz transition. We should also note that experimentally determined $\gamma$ values can be affected by the inhomogeneities in investigated films^32^. Accordingly, the inhomogeneities may lead to the ${\phi }_{B}=0$ value determined from the experiment, even when SdH oscillations arise from DSM regions with ${\phi }_{B}=-1$/2.

Thus, the SC state in films under study may coexists with the DSM phase, implying that it can be of topological nature, which is also supported by the arguments reported for mangetron-sputtered films^20^. Due to the difference of SC parameter values (${H}_{c}$ and ${T}_{c}$) for the films synthesized by different methods, we believe that the emergent SC state can be tuned further not only by the variation of synthesis conditions, but also by applying external stimuli (e.g., pressure or gate voltage). Therefore, the investigated polycrystalline Cd_3_As_2_ films can be used as a platform for studies of topological SC and other related phenomena, e.g., the interaction between SC state and unconventional surface carriers.

## Conclusions

We report the realization of reproducible superconductivity in Cd_3_As_2_ thin films without application of any external factors. The observed SC state shows the same features as the ones previously observed in SC magnetron-sputtered Cd_3_As_2_ films. It clearly reveals the intrinsic nature of SC in studied systems, which cannot be attributed to any particular growth method, while the latter affects the parameter values of the SC state. Moreover, we observed SdH oscillations in high magnetic fields. We argue that these oscillations, in combination with detected Cd_3_As_2_ tetragonal phase, indicate preservation of the DSM phase in studied films. This DSM phase may be partially suppressed due to the Lifshitz transition of electron spectrum. Thus, investigated Cd_3_As_2_ films, hosting both a SC state and the DSM phase, can be used as a fruitful playground to realize and study topological superconductivity.

## Methods

Studied Cd_3_As_2_ films were synthesized using the thermal evaporation technique in a high-vacuum (<10${}^{-4}$ Pa) chamber. Single crystals of Cd_3_As_2_ used for the film synthesis were grown by directional solidification in a vertical furnace from a polycrystalline Cd_3_As_2_ precursor. The growth procedure was similar to that described earlier^35^. For the film growth we used (111) Si substrates with a thin native oxide layer and deposited Ag contacts. The dimensions of used substrates were 3 × 5 × 0.6 mm. During the deposition process, the substrates were kept at room temperature. Samples were shaped as a Hall bar with conducting channel length $L\approx 2$ mm and width $W\approx 0.7$ mm. In this work we studied two samples (labeled A and B), synthesized with similar deposition parameters, showing the degree of reproducibility of films properties. Thickness of studied films was about 100 nm, that corresponds to the average growth rate of about 20 nm per minute.

SEM images were obtained using Scios (ThermoFisher Scientific, USA) dual beam instrument equipped with EDX spectrometer (EDAX, USA). The XRD patterns of the studied films were obtained at room temperature using a Bruker D8 Advance diffractometer (Bruker, Germany) with a Cu ${K}_{\alpha }$ radiation source ($\lambda =1.54$ Å, $U=40$ kV, $I=40$ mA). The patterns were recorded in the range of $2\theta =1{0}^{\circ }-9{0}^{\circ }$ with 0.014${}^{\circ }$ steps and an exposure time of 3 s per point. Obtained X-ray diffraction patterns were processed using ICDD PDF-2 powder references. Transport measurements were conducted using a 20-T superconducting magnet with a dilution refrigerator (SCM1) at the National High Magnetic Field Laboratory (NHMFL), Tallahassee, Florida, USA. Magnetoresistance was measured in a standard four-probe configuration using SR860 lock-in amplifiers (Stanford Research Systems, USA) with a low-frequency measurement current of about 100 nA. Measurements of the differential resistance $dV$/$dI$ were performed utilizing a current source Model No. 6221 and a nanovoltmeter Model No. 2182A (Tektronix, Inc., USA). Here we present data measured in two field orientations with respect to the film plane - transverse (${B}^{\perp }$) and longitudinal (${B}^{\parallel }$). In the latter case, the magnetic field was parallel to the measurement current direction.
